# Inhibitory Properties of Aqueous Ethanol Extracts of Propolis on Alpha-Glucosidase

**DOI:** 10.1155/2015/587383

**Published:** 2015-02-12

**Authors:** Hongcheng Zhang, Guangxin Wang, Trust Beta, Jie Dong

**Affiliations:** ^1^Bee Research Institute, Chinese Academy of Agricultural Sciences, Beijing 100093, China; ^2^National Research Center of Bee Product Processing, Ministry of Agriculture, Beijing 100093, China; ^3^Department of Food Science, University of Manitoba, Winnipeg, MB, Canada R3T 2N2

## Abstract

The objective of the present study was to evaluate the inhibitory properties of various extracts of propolis on alpha-glucosidase from baker's yeast and mammalian intestine. Inhibitory activities of aqueous ethanol extracts of propolis were determined by using 4-nitrophenyl-D-glucopyranoside, sucrose and maltose as substrates, and acarbose as a positive reference. All extracts were significantly effective in inhibiting *α*-glucosidase from baker's yeast and rat intestinal sucrase in comparison with acarbose (*P* < 0.05). The 75% ethanol extracts of propolis (75% EEP) showed the highest inhibitory effect on *α*-glucosidase and sucrase and were a noncompetitive inhibition mode. 50% EEP, 95%, EEP and 100% EEP exhibited a mixed inhibition mode, while water extracts of propolis (WEP) and 25% EEP demonstrated a competitive inhibition mode. Furthermore, WEP presented the highest inhibitory activity against maltase. These results suggest that aqueous ethanol extracts of propolis may be used as nutraceuticals for the regulation of postprandial hyperglycemia.

## 1. Introduction

Diabetes mellitus is the most common endocrine disorder disease caused by inherited or acquired deficiency in insulin excretion and by decreased responsiveness of the organs to secreted insulin. According to the recent reports, the number of diabetic patients in the world would amount to 366 million in 2030 and death related to diabetes mellitus accounts for about 9% global mortality, particularly type 2 diabetes mellitus [1]. In China, more than 92 million adults have diabetes, and 95 percent of them are type 2 diabetes mellitus, according to the survey of current epidemiology [2]. Because of more-affluent lifestyles, the number of people with diabetes is predicted to increase in the future. Patients with type 2 diabetes mellitus usually tend to have long-term complications, such as retinopathy, cataract, atherosclerosis, neuropathy, nephropathy, and impaired wound healing [3]. Diabetes mellitus is characterized by abnormally high plasma glucose. Hyperglycemia has played a central role in pathogenesis of complications related to diabetes mellitus. In treatment of type 2 diabetes, suppression of postprandial hyperglycemia can decrease the risk of those complications. Hence, control of postprandial hyperglycemia should be a primary goal in the prevention and management of type 2 diabetes.

Alpha-glucosidases are a series of enzymes, including sucrase and maltase, located in the brush-border surface of intestinal cell, which catalyze the final step in the digestive process of carbohydrates to release absorbable monosaccharides resulting in increased blood glucose levels [4]. If *α*-glucosidases are inhibited, the liberation of D-glucose from dietary complex carbohydrates can be retarded. Thus, *α*-glucosidase inhibitors have become candidates of hot pursuits to delay the digestion and absorption of carbohydrates and restrain postprandial hyperglycemic excursions. Alpha-glucosidase inhibitors as one of therapeutic approaches for diabetes mellitus have been known since the early 1990s [5]. At present, some *α*-glucosidase inhibitors, such as acarbose, miglitol, and voglibose, have been approved for clinical use in the management of type 2 diabetes, as well as the treatment of obesity. However, some synthetic *α*-glucosidase inhibitors have side-effects, such as flatulence, diarrhea, and abdominal cramping, all of which are associated with incomplete carbohydrate absorption [6]. As a result, many researchers have focused on novel *α*-glucosidase inhibitors from natural materials, which are used to develop functional foods or lead compounds for antidiabetic treatment including* Syagrus romanzoffiana*,* Adhatoda vasica Nees*, and* Syzygium cumini *(Linn.) seed kernel [7–9]. Some medicinal plant species have more potent *α*-glucosidase inhibitory activities than powerful synthetic *α*-glucosidase inhibitors such as acarbose [10].

Propolis is a colored and aromatic colloidal substance collected by honeybees through adding their saliva secreted to the resinous plant exudate, which is used to build honeycomb and to fight against the invasion of pathogenic microorganism. The chemical composition of propolis varies and depends mainly upon the local flora in the region of collection. At present, more than 300 components have been identified including flavonoids, phenolic acids, alcohols and their esters, ketones, and inorganic compounds [11]. Propolis has been applied in popular folk medicine since 3000 BC due to possessing a broad spectrum of biological activities such as antioxidant, antimicrobial, anti-inflammatory, antiviral, anticancer, and antihepatotoxic properties [12]. Recent studies show that ethanol and water extracts of propolis can control the glycemia and modulate glucose and lipid metabolism in STZ-induced diabetic rats [13, 14]. Ethanolic extracts of Brazilian green propolis were also shown to possess therapeutic potential in STZ-induced diabetic rats [15]. In China, propolis has been approved for use in functional foods carrying a health claim of controlling glycemia in 1999 by the Ministry of Health [16]. Moreover, propolis has been accepted as adjuvant therapy drugs for diabetes mellitus in Chinese Pharmacopoeia in 2005 [17]. However, there are few reports whether controlling glycemia of propolis is related to its inhibitory activities against *α*-glucosidase.

In the present study, we assessed inhibitory effects of aqueous ethanol extracts of propolis on alpha-glucosidase and the kinetics of enzyme inhibition by using the Michaelis-Menten model.

## 2. Material and Methods

### 2.1. Chemicals and Reagents

Alpha-glucosidases (EC 3.2.1.20) from baker's yeast, rat intestinal acetone powder, p-nitrophenyl-*α*-D-glucopyranoside (PNP-glucoside), sucrose, maltose, piperazine-1,4-bisethanesulfonic acid (PIPES), Folin-Ciocalteu, and Glucose-Trinder 100 were obtained from Sigma Chemical Co. (St. Louis, MO, USA). Dimethyl sulphoxide (DMSO, cell culture grade) was purchased from Applichem Co. (Darmstadt, Germany). The crude propolis was procured from Bee Research Institute, Chinese Academy of Agricultural Science (Beijing, China). All reagents were of analytical grade.

### 2.2. Preparation of Aqueous Ethanol Extracts of Propolis

Propolis extracts were prepared as described by Huang et al. [18] with slight modification. Crude propolis (10 grams) was, respectively, extracted with 200 mL aqueous ethanol solvent at concentrations ranging from 0%, 25%, 50%, 75%, and 95% to 100% (in water, v/v) by using ultrasonic extract for 4 hours (ultrasonic extractor, DCTZ-1000, Beijing Hongxianglong Biotechnol Co. Ltd., Beijing, China). The suspensions were centrifuged at 12,000 ×g for 30 min to obtain supernatants which were concentrated at 45°C in a rotary evaporator (Rotavapor, R-215, Buchi Co., Ltd., Switzerland) and then freeze-dried. Extracts were stored in zip lock bag at 4°C in darkness. The extracts were dissolved by DMSO until application.

### 2.3. Determination of Total Phenolic Contents

Total polyphenol contents in propolis extracts were determined according to the Folin-Ciocalteu colorimetric method with slight modifications [19]. A standard curve was built with gallic acid solution. Aliquots ranging from 0 to 1.2 mL of standard solution (100 *μ*g/mL) were pipetted into 10 mL volumetric flasks containing 6 mL distilled water. 0.5 mL of 2 mol/L Folin-Ciocalteu solution and 1.5 mL of 10% Na_2_CO_3_ (w/v) were added and the volume made up to 10 mL with distilled water. Following mixing, the solution was measured at 765 nm after 10 min by using UV-2500/Ultraviolet visible spectrophotometer with UVProbe software (Shimadzu Co., Ltd., Tokyo, Japan). The blank was also prepared without addition of the standard aliquots. For determination of the total phenolic contents in propolis extracts, 20 *μ*L of aqueous solution at a concentration of 1 mg/mL was used. Total phenolic contents were expressed as milligrams gallic acid equivalents per gram extract (mg GAE/g).

### 2.4. Determination of Total Flavonoid Contents

Contents of flavonoid in propolis extracts were determined according to the method of Kaijv et al. [20], with minor modifications. A standard curve was built with chrysin solution. Aliquots ranging from 0 to 12 mL of standard solution (100 *μ*g/mL) were pipetted into 25 mL volumetric flasks containing 1.0 mL aliquots of 5% NaNO_2_ (w/v) solution. After 6 min, 1.0 mL 10% AL(NO_3_)_3_ (w/v) solution was added and thoroughly mixed. 10 mL 4.3% NaOH (w/v) solution was added after 6 min and the volume was made up with ethanol. The blank was prepared by using ethanol instead of standard solution or sample solutions. After 15 min, the absorbance was measured at 510 nm by using UV-2500/Ultraviolet visible spectrophotometer with UVProbe software (Shimadzu Co., Ltd., Tokyo, Japan). For determination of the total flavonoid contents in propolis extracts, 0.5 mL of aqueous solution at a concentration of 1 mg/mL was used. Total flavonoid contents were expressed as milligrams chrysin equivalents per gram extract (CE).

### 2.5. Inhibitory Activity Assay for Baker's Yeast Alpha-Glucosidase

Alpha-glucosidase inhibitory effect of propolis extracts was assayed according to the procedure described previously by Kim et al. [21] with slight modifications. The enzyme reaction was performed using PNP-glycoside as a substrate in 0.1 M PIPES buffer (pH 6.8). 1 mL PNP-glycoside (1.0 mM) was premixed with 0.1 mL propolis extracts at various concentrations (2 *μ*g/mL–200 *μ*g/mL) in 2.05 mL PIPES buffer and the reaction system was preheated at 37°C for 30 min. Then, 0.5 mL enzyme solution (0.11 U/mL) was added into mixture and absorbances were immediately measured at 30-second intervals for 15 min at 415 nm using UV-2500/Ultraviolet visible spectrophotometer with UVProbe software (Shimadzu Co., Ltd., Tokyo, Japan). The inhibition activity was calculated by the equation: Inhibition  (%) = [1 − (*A*  sample/*A*  control)] × 100% [21]. Acarbose was also assayed as a positive reference. Concentrations of extracts resulting in 50% inhibition of enzyme activity (IC_50_ values) were determined graphically. Different propolis extracts were compared on the basis of their IC_50_ values estimated from the dose response curves.

### 2.6. Inhibitory Activity Assay for Rat Intestinal Sucrase

Rat intestinal sucrase inhibitory effects of propolis extracts were assayed according to a procedure described previously [22] with slight modifications. Briefly, 100 mg rat intestinal acetone powder was homogenized in 15 mL 0.9% NaCl solution (w/v). After centrifugation at 12,000 g for 40 min, the supernatant was stored at 4°C as enzyme solution. 0.8 mL 56 mmol/L sucrose and 0.75 mL 0.1 M PIPES buffer (pH 7.0) were premixed with 0.1 mL propolis extracts of various concentrations (2 *μ*g/mL–200 *μ*g/mL). After preincubating for 5 min at 37°C, 0.7 mL of the enzyme solution was added and the reaction was carried out at 37°C for 30 min. The reaction was terminated by adding 0.75 mL of 2 mol/L Tris-HCl buffer (pH 6.9). For the blank, 0.1 mL DMSO was used in place of propolis extract. The concentration of glucose released from the reaction mixtures was determined colorimetrically using Glucose-Trinder 100 [23] and the absorbance measured at 505 nm after incubation at 37°C for 20 min. The inhibition activity was calculated by the equation: Inhibition  (%) = [1 − (*A*  sample/*A*  control)] × 100%. Acarbose was also assayed as a positive reference.

### 2.7. Inhibitory Activity Assay for Rat Intestinal Maltase

Rat intestinal maltase inhibitory effect of propolis extracts was assayed according to a procedure described previously [24]. The assay for rat intestinal maltase inhibitory activity was carried out in a similar manner as the rat intestinal sucrase inhibitory activity, except that 10 mM maltose in 0.1 M PIPES buffer (pH 7.0) was used as a substrate. Acarbose was also assayed as a positive reference.

### 2.8. Inhibition Kinetics on Alpha-Glucosidase Activity

Inhibition model of different extracts of propolis on baker's yeast *α*-glucosidase was tested according to procedure described previously [4] with minor modifications. Alpha-glucosidase activity was measured with increasing concentrations of PNP-glucoside (0.4–6.0 mM) in the absence or presence of propolis extracts at various concentrations (0.1–2.0 mg/mL). The reaction was initiated by addition of enzyme and the reaction measured at 415 nm at 30-second interval for 15 min. The enzyme assay data containing various concentration of PNP-glucoside and propolis extracts were used to construct the Lineweaver-Burk plots to determinate inhibition model, *V*_max⁡_ and Km values.

### 2.9. Inhibition Constant Analysis

The inhibition constant of propolis on alpha-glucosidase was calculated by Ki = [*I*]/(*α* − 1), where [*I*] represents the concentration of inhibitor, and *α* is confirmed by *v* = (*V*_max⁡_/*α*[*S*])/(Km + [*S*]). *V*_max⁡_ and Km values were obtained from the above assay, and [*S*] represents the concentration of substrate.

### 2.10. Statistical Analysis

Experimental results were expressed as mean ± standard deviation (SD) of triplicate measurements. The data were subjected to one way analysis of variance (ANOVA). Significance differences at *P* < 0.05 among treatment means were obtained using Duncan's multiple range test using SAS version 9.1 (SAS Institute Inc., Cary, NC, USA).

## 3. Results and Discussion

### 3.1. Total Phenolic and Flavonoid Contents of Aqueous Ethanol Extracts of Propolis

Phenolics are the predominant bioactive materials in propolis which have been reported to have multiple biological effects, including antidiabetes. Therefore, measurement of total phenolic contents (TPC) and total flavonoid contents (TFC) was inevitable. Total phenolic and flavonoid contents in various aqueous ethanol extracts of propolis are presented in Table 1. TPC ranged from 273.94 to 386.49 mgGAE/g extracts increasing in the following order: 25% EEP > WEP > 50% EEP > 75% EEP > 95% EEP > 100% EEP. TPC was not significantly different among WEP, 25% EEP, and 50% EEP. TFC ranged from 352.32 to 697.36 mg CE/g extracts increasing in the following order: 75% EEP > 100% EEP > 95% EEP > 50% EEP > 25% EEP > WEP. TFC was not significantly different among 100% EEP, 95% EEP, and 75% EEP.

The total phenolic and flavonoid contents of propolis extracts varied with different concentrations of hydrous ethanol. A similar report shows that ethanol/water concentrations correlate with the amount and composition of phenolic compounds and flavonoids of propolis extracts [25]. Moreover, propolis from various areas of China was found to contain a wide variety of bioactive compounds, mainly phenolic acids and flavonoids [26]. In the current study, while ethanol concentrations in hydrous ethanol were less than 50% as extraction solvent, the TPC of these extracts were significantly higher than those containing higher ethanol concentrations (*P* < 0.05). These propolis extracts may mainly contain more hydrophilic phenolic compounds, cinnamic acid derivatives [27]. On the other hand, when ethanol concentrations were higher than 50%, TFC of the extracts were significantly higher compared to those with lower ethanol concentrations (*P* < 0.05). These propolis extracts mainly contain a significant increase in the ratio of more hydrophobic flavonoid compounds, such as apigenin, kaempferol, and chrysin [26].

### 3.2. Inhibition of Aqueous Ethanol Extracts of Propolis against Alpha-Glucosidase

The *α*-glucosidase inhibitory activity of various propolis extracts is shown in Table 2. The propolis extracts had ever been found to be reversibly bound to *α*-glucosidase in our previous report. The effectiveness of enzymatic inhibition of the various extracts was determined by calculating IC_50_. The lower the value showed, the higher the enzymatic inhibition. IC_50_ values of propolis extracts ranged from 7.24 to 20.01 *μ*g/mL against baker's yeast *α*-glucosidase and from 32.34 to 53.12 *μ*g/mL against rat intestinal sucrase. These values were significantly (*P* < 0.05) lower than 177.5 *μ*g/mL and 538.3 *μ*g/mL of acarbose, a positive reference, respectively. Thus, all propolis extracts possessed much stronger inhibitory effects on *α*-glucosidase from baker's yeast and sucrase compared to acarbose. The 75% EEP showed the highest inhibitory effect on *α*-glucosidase from baker's yeast and sucrase, and IC_50_ values accounted for only 4% and 6% of that of acarbose, respectively. For rat intestinal maltase, WEP had the highest inhibitory activity among all extracts with IC_50_ value of 32.67 *μ*g/mL that was significantly higher than that of acarbose of 22.5 *μ*g/mL (*P* < 0.05). All propolis extracts showed weak inhibitory effects on maltase in comparison to acarbose.

Many plant extracts from food and Chinese traditional medicine have been reported to have antidiabetic activity [5]. These antidiabetic phytochemicals are probably comprising phenolic compounds, such as flavonoids and phenolic acids [28]. Propolis extracts contain phenolic compounds which are classified into two major categories, phenolic acids and flavonoids. As shown in Table 1, TPC and TFC of various ethanol extracts of propolis were different. Similarly, the inhibitory effects of various propolis extracts on alpha-glucosidases were also different (Table 2). The 75% EEP possessed the highest flavonoid contents and the strongest inhibitory effect on *α*-glucosidases from baker's yeast and rat intestinal sucrase among all extracts. Wang et al. [29] also reported that some flavonoids have the higher inhibitory effect on rat sucrase than that of rat maltase. Moreover, the study revealed WEP and 25% EEP had higher TPC and stronger inhibitory effects on rat intestinal maltase among all extracts. Some phenolics such as chebulanin, chebulagic acid, and chebulinic acid were reported to have the same potential against rat intestinal maltase [30]. Kamiyama et al. [31] similarly found that some catechin derivatives had better inhibitory activity against rat intestinal maltase than rat intestinal sucrase. Therefore, it seems to assume that extracts with higher TFC could have better inhibition against rat intestinal sucrase; similarly extracts with higher TPC could possess better inhibition against rat intestinal maltase.

### 3.3. Inhibitory Kinetics of Aqueous Ethanol Extracts of Propolis against Baker's Yeast Alpha-Glucosidase

Yeast *α*-glucosidase is readily available in a pure form and has been widely used for antidiabetic nutraceutical and medicinal investigations as a model for screening potential inhibitors and studying inhibitory mechanism [5]. To find the inhibition mechanism of aqueous ethanol extracts of propolis, inhibitory kinetics against yeast *α*-glucosidase was analyzed by Lineweaver-Burk plots using data derived from enzyme assay containing different concentrations of PNP-glucoside in the absence or presence of different concentration of inhibitor.

**Figure 1:**
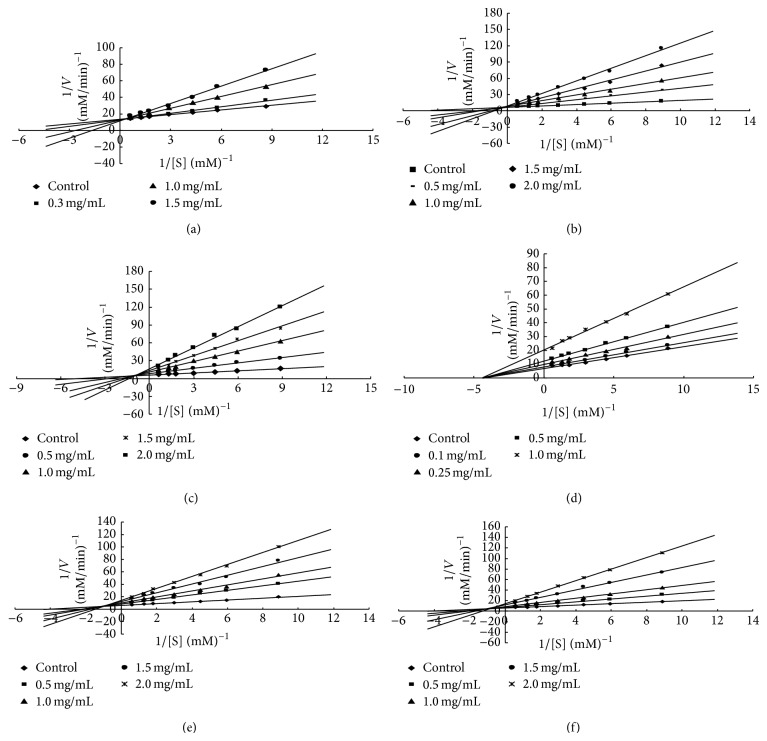
Lineweaver-Burk plots of inhibition kinetics of yeast alpha-glucosidase inhibitory effects by WEP (a), 25% EEP (b), 50% EEP (c), 75% EEP (d), 95% EEP (e), and 100% EEP (f). Water extracts of propolis were expressed as WEP. Extracts of propolis using 25%, 50%, 75%, 95%, and 100% (in water, v/v) aqueous ethanol solvents were expressed as 25% EEP, 50% EEP, 75% EEP, 95% EEP, and 100% EEP, respectively.

As can be seen in Table 3, various aqueous ethanol extracts of propolis had different inhibition modes. Double-reciprocal plots of enzyme kinetics demonstrated that the inhibition of WEP and 25% EEP was competitive inhibition mode (Figures 1(a) and 1(b)), and the Ki values were 10.43 *μ*g/mL and 9.85 *μ*g/mL (Table 3). Plots also indicated that the type of 50% EEP, 95% EEP, and 100% EEP was mixed inhibition mode (Figures 1(c), 1(e), and 1(f)), and the Ki values were 9.92 *μ*g/mL, 9.65 *μ*g/mL, and 10.89 *μ*g/mL, respectively (Table 3). Moreover, inhibitory mode of 75% EEP was noncompetitive inhibition (Figure 1(d)), and the Ki value was 15.18 *μ*g/mL, a value significantly higher than those of other extracts (*P* < 0.05).

Phenolic compounds are able to inhibit the activities of carbohydrate-hydrolysing enzymes due to their ability to bind with proteins [32]. As can be seen, different aqueous ethanol extracts of propolis were revealed to have different inhibition modes against *α*-glucosidase. Probably that is because phenolic compounds in different EEP have different bound modes with *α*-glucosidase. The inhibition of WEP and 25% EEP was a competitive mode characterized by the fact that the substrate and inhibitor compete for the same binding site in the enzyme, the so-called active site or substrate-binding site. It indicated that phenolic compounds in WEP and 25% EEP can bind to the active site of *α*-glucosidase. The inhibition of 75% EEP was a noncompetitive mode in that inhibitor is not binding to the active site on *α*-glucosidase. Inhibition mode was a mixed inhibition for 50% EEP, 95% EEP, and 100% EEP. For the noncompetitive and mixed inhibition mode, the binding of inhibitor can influence the binding of substrate by changing the conformation of the enzyme. Different inhibition modes shown by various EEP were likely due to the different bioactive compounds in the extracts. 75% EEP was found to contain the highest TFC among EEP and exhibit noncompetitive inhibition. TFC of 95% EEP and 100% EEP were lower but not significantly different than 75% EEP and showed mixed inhibition. A similar trend was observed that many flavonoids exhibited mixed or noncompetitive type of inhibition [28, 33]. WEP and 25% EEP contained the higher TPC and exhibited a competitive inhibition mode. The ethanol extract of* G. montanum* rich in phenolic composition also showed competitive inhibition against yeast *α*-glucosidase [34]. It seems to assume that inhibition of aqueous ethanol extracts of propolis with the higher TPC is likely a competitive mode while those with higher TFC tend to have a noncompetitive or mixed mode.

All aqueous ethanol extracts of propolis showed stronger inhibition against the yeast *α*-glucosidase compared to acarbose, especially for those extracts using the higher concentration of ethanol as the solvent. It is probably that the ability to bind to wide regions of enzyme other than the active site enables these propolis extracts as noncompetitive or mixed inhibitors a broader specificity of inhibition, compared with acarbose, a competitive inhibitor. Another advantage of aqueous ethanol extracts of propolis over acarbose is that these propolis extracts, noncompetitive or mixed inhibitors, may not be affected by the higher concentration of the substrate in contrast to acarbose which is a competitive inhibitor. It is reported that, with higher carbohydrate food intake, higher concentrations of acarbose as a competitive inhibitor would be needed to show the same effect. For mixed inhibition, the inhibitor would be still effective at lower concentrations [6].

## 4. Conclusion

In the present study, all aqueous ethanol extracts of propolis show stronger inhibitory effects on yeast *α*-glucosidase and rat intestinal sucrose than that of acarbose as a positive reference. Our findings seem to prove that the controlling glycemia of propolis may be related to the inhibitory activities against *α*-glucosidase. Thus, aqueous ethanol extracts of propolis may be used as nutraceuticals for the regulation of postprandial hyperglycemia. For further study, the fractions from propolis having inhibitory activity against *α*-glucosidase are being purified and their chemical structures are being characterized.

## Figures and Tables

**Table 1:** Total phenolic and flavonoid contents of various ethanol extracts of propolis.

Aqueous ethanol extracts of propolis (EEP)	Total phenolic contents (mg GAE/g extracts)	Total flavonoid contents (mg CE/g extracts)
Water extracts of propolis (WEP)	369.31 ± 26.87^ab^	352.32 ± 0.67^a^
25% ethanol extracts of propolis (25% EEP)	386.49 ± 17.36^a^	504.18 ± 32.10^b^
50% ethanol extracts of propolis (50% EEP)	355.71 ± 31.35^ab^	620.80 ± 4.08^c^
75% ethanol extracts of propolis (75% EEP)	341.05 ± 14.89^b^	697.36 ± 6.15^d^
95% ethanol extracts of propolis (95% EEP)	312.65 ± 24.13^cb^	637.84 ± 8.13^cd^
100% ethanol extracts of propolis (100% EEP)	273.94 ± 1.76^d^	673.24 ± 5.96^d^

Note: Water extracts of propolis were expressed as WEP. Extracts of propolis using 25%, 50%, 75%, 95%, and 100% (in water, v/v) aqueous ethanol solvents were expressed as 25% EEP, 50% EEP, 75% EEP, 95% EEP, and 100% EEP, respectively. TPC was expressed as milligram of gallic acid equivalent per gram of propolis extracts (mg GAE/g extracts). TFC was expressed as milligram of rutin equivalent per gram of propolis extracts (mg RE/g extracts). Dates are mean ± standard deviation (*n* = 3). Values in the same column followed by the same lower case letter are not significantly different by Duncan's multiple range test (*P* < 0.05).

**Table 2:** Inhibition of propolis extracts against yeast and rat intestinal alpha-glucosidase.

Aqueous ethanol extracts of propolis (EEP)	IC_50_ (*μ*g/mL)		
	Baker's yeast alpha-glucosidase	Rat intestinal sucrase	Rat intestinal maltase
WEP	16.0 ± 1.38^bc^	37.91 ± 0.52^ab^	32.67 ± 0.24^e^
25% EEP	11.32 ± 2.23^ab^	40.25 ± 0.97^ab^	44.80 ± 0.25^d^
50% EEP	12.90 ± 1.14^b^	53.12 ± 0.95^b^	100.60 ± 2.95^b^
75% EEP	7.24 ± 1.16^a^	32.34 ± 0.61^a^	71.82 ± 2.95^c^
95% EEP	16.26 ± 0.43^bc^	32.91 ± 0.54^a^	102.76 ± 1.47^b^
100% EEP	20.01 ± 1.54^c^	38.06 ± 0.56^ab^	131.12 ± 1.83^a^
Acarbose	177.47 ± 6.28^d^	538.30 ± 26.69^c^	22.46 ± 0.86^f^

Note: Water extracts of propolis were expressed as WEP. Extracts of propolis using 25%, 50%, 75%, 95%, and 100% (in water, v/v) aqueous ethanol solvents were expressed as 25% EEP, 50% EEP, 75% EEP, 95% EEP, and 100% EEP, respectively. Dates are mean ± standard deviation (*n* = 3). Values in the same column followed by the same lower case letter are not significantly different by Duncan's multiple range test (*P* < 0.05). Inhibition of propolis extracts against yeast and rat intestinal alpha-glucosidase were both expressed as IC_50 _(concentration of total phenolics able to scavenger 50% of alpha-glucosidase activity).

**Table 3:** Inhibitory kinetics and Ki values of various propolis extracts against baker's yeast alpha-glucosidase.

Aqueous ethanol extracts of propolis (EEP)	Kinetic mode	Ki (*μ*g/mL)
WEP	Competitive inhibition	10.43 ± 1.41^b^
25% EEP	Competitive inhibition	9.85 ± 2.48^b^
50% EEP	Mixed inhibition	9.92 ± 0.43^b^
75% EEP	Noncompetitive inhibition	15.18 ± 0.58^a^
95% EEP	Mixed inhibition	9.65 ± 1.54^b^
100% EEP	Mixed inhibition	10.89 ± 2.84^b^

Note: Water extracts of propolis were expressed as WEP. Extracts of propolis using 25%, 50%, 75%, 95%, and 100% (in water, v/v) aqueous ethanol solvents were expressed as 25% EEP, 50% EEP, 75% EEP, 95% EEP, and 100% EEP, respectively. The inhibition constant of propolis on alpha-glucosidase was expressed as Ki. Dates are mean ± standard deviation (*n* = 3). Values in the same column followed by the same lower case letter are not significantly different by Duncan's multiple range test (*P* < 0.05).
